# Classification Algorithms for Early Tooth Demineralization Assessment by Impedance Spectroscopy

**DOI:** 10.3390/s25113476

**Published:** 2025-05-31

**Authors:** Isabella Sannino, Luca Lombardo, Leila Es Sebar, Marco Parvis, Allegra Comba, Nicola Scotti, Emma Angelini, Leonardo Iannucci, Tolou Shokuhfar, Sabrina Grassini

**Affiliations:** 1 Department of Applied Science and Technology, Politecnico di Torino, 10129 Turin, Italy; leila.essebar@polito.it (L.E.S.); emma.angelini@polito.it (E.A.); leonardo.iannucci@polito.it (L.I.); sabrina.grassini@polito.it (S.G.); 2 Department of Electronics and Telecommunications, Politecnico di Torino, 10129 Turin, Italy; luca.lombardo@polito.it (L.L.); marco.parvis@polito.it (M.P.); 3 Department of Surgical Science, Università degli Studi di Torino, 10124 Turin, Italy; allegra.comba@unito.it (A.C.); nicola.scotti@unito.it (N.S.); 4 Department of Biomedical Engineering, University of Illinois, Chicago, IL 60607, USA; tolou@uic.edu

**Keywords:** impedance spectroscopy, multi-layer perception, tooth demineralization, tooth lesion classifiers, carious lesions

## Abstract

Oral caries is one of the most common oral diseases worldwide, affecting about 2.4 billion people. This phenomenon always starts with enamel demineralization, eventually progressing to tooth cavitation and loss when not properly treated. Nowadays, the standard diagnostic techniques to detect demineralization strongly depend on the operator’s expertise and are characterized by fairly low sensitivity and specificity, and/or involve ionizing radiation. This study investigates the feasibility of a non-invasive, effective, rapid, and radiation-free approach employing impedance spectroscopy for early caries detection. Two binary classifiers were developed for automated assessment and validated using a dataset obtained by in vitro demineralization of human teeth. A computationally efficient single-neuron classifier, utilizing a single impedance phase measurement at 15 Hz, achieved 88% accuracy, offering a lightweight, low-power solution suitable for microcontroller implementation and rapid measurements. A Multi-Layer Perceptron (MLP) classifier, utilizing equivalent circuit element values, yielded a similar accuracy of 86%. A prototype of a diagnostic portable tool was developed and characterized, demonstrating reliable impedance phase measurement (uncertainty < 2°). The performance of these classifiers meets or exceeds the existing AI-based methodologies for caries detection relying on radiographic data. This work introduces a novel application of AI to tooth impedance spectra, addressing a significant research gap in non-invasive diagnostics and laying the foundation for a novel, accessible, and accurate tool for early caries management.

## 1. Introduction

Oral diseases are a significant public health problem, affecting nearly 3.5 billion people globally, as per the global oral health status report in 2023 [1]. These diseases can cause stress, pain, or infections, significantly lowering people’s quality of life. Unfortunately, treatments for oral health conditions are quite expensive, and they are usually not covered under universal health coverage. The financial burden of oral diseases has been estimated to be around USD 387 billion in direct costs and USD 323 billion in indirect costs. Thus, oral diseases represent a major concern for public health [2]. The World Health Organization suggests that dental caries, affecting 2.4 billion people, is one of the most prevalent oral diseases worldwide, affecting 60% to 90% of school-aged children, whereas periodontal disease affects 14% of middle-aged adults [3]. The term ‘dental caries’ or ‘caries’ refers to both the carious lesion (cavitated or non-cavitated) and the caries process [4]. It is a multi-factorial disease that gradually erodes dental hard tissues through the activity of endogenous bacteria. The latter are present on the tooth’s natural surface within the bacterial biofilm, commonly referred to as dental plaque. Oral plaque bacteria, which naturally occur in the oral cavity, ferment carbohydrates and sugars present in foods and drinks, producing weak organic acids that demineralize tooth tissues. The process starts on the external part of the dental crown, the enamel, which is primarily composed of mineral calcium phosphate arranged in a crystalline structure known as hydroxyapatite (HA), with the formula Ca_10_(PO_4_)_6_(OH)_2_. Acid exposure leads to enamel demineralization through the precipitation of calcium and phosphate from the tightly packed hydroxyapatite lattice, thus starting the carious process. Regardless of tooth type or location, all caries start with demineralization.

Demineralization removes essential mineral ions from tooth hard tissue, weakening its structure. However, this process is reversible and lost ions can be restored to HA crystals through remineralization treatments or naturally. Studies show that demineralization and remineralization continuously occur throughout the day, influenced by oral pH and the availability of saliva mineral ions [5]. After enamel damage, the process progresses on the underlying layers (dentin and cementum) up to irreversible damage of the tooth and consequent cavitation. Therefore, besides the adoption of preventive healthcare strategies, the availability of effective non-invasive methods for early demineralization assessment is a crucial aspect.

In clinical practice, the primary method for identifying pathologic conditions affecting the oral cavity is through visual assessment by expert dentists. The diagnosis of caries, particularly, is performed using visual and visual–tactile tools, i.e., dental radiographs and sharp explorers [6]. These tools help to identify alterations in the appearance of teeth in enamel and dentin after a demineralization process. However, these clinical diagnostic methods are strongly operator-dependent and carry potential risks due to ionizing radiation exposure. Furthermore, these methods show low sensitivity and specificity, making them less effective in detecting carious lesions at an early stage [7,8].

The availability of automatic non-invasive diagnostic systems able to assess tooth demineralization at its early stage independently from the expertise of the operator may, therefore, represent an extremely valuable tool to aid dentists’ diagnoses. Indeed, in the current era of big data, the adoption of machine learning (ML) techniques has gained remarkable ground in various fields, such as finance, robotics, speech recognition, spatial science, and many others. Moreover, ML has achieved promising results in the medical and dental industries. Indeed, the employment of ML-based image analysis in dermatology, ophthalmology, and radiology has achieved accuracy levels comparable to those of experienced clinicians. In particular, deep learning (DL) techniques, e.g., Convolutional Neural Networks (CNNs), have found wide application in dentistry for identifying either specific regions or pathologic conditions within digital images, including photographs, bitewings, periapical radiographs, panoramic X-rays, and dental cone-beam computed tomography [9,10]. In the field of endodontics, CNNs have been employed to interpret the morphology of first molar roots and detect abnormalities in panoramic radiographs with promising results [11].

ML and DL algorithms have shown promising results in detecting and diagnosing dental caries [12]. These algorithms analyze a large number of dental images to recognize patterns and characteristics associated with caries, facilitating precise detection in novel unseen images [13]. An automatic caries detection and segmentation system, based on CNN algorithms applied to dental bitewing radiographs, achieved a sensitivity of 0.84 and precision of 0.81 for caries detection in an internal dataset. However, the performance slightly declined in an external dataset, with sensitivity and precision values of 0.77 and 0.78, respectively [14]. CNN models, trained on smaller datasets utilizing different strategies (i.e., image recognition, edge extraction, and image segmentation), demonstrated good performance in detecting proximal caries on periapical radiographs. These models achieved area under the receiver operating characteristic curve (AUC) values of 0.805, 0.860, and 0.549, respectively [15]. The DL models, particularly U-Net, showcased improved performance in segmenting tooth caries when trained with larger datasets of bitewing radiographs. Cantu et al. [16] achieved an accuracy of 0.80, sensitivity of 0.75, and specificity of 0.83 with their U-Net model. Furthermore, CNNs integrated with long short-term memory models, as utilized by Prena et al. [17], reached an accuracy of 93% in detecting and diagnosing dental caries on periapical dental images. Multi-Layer Perceptron (MLP) models were trained for caries detection, achieving notable accuracies in dental intraoral radiographs. Rodríguez et al. [18] reported a detection accuracy of 89%, while Rad et al. [19] achieved an accuracy performance of 90%.

Nonetheless, some studies also explored alternative clinical techniques, such as Near-Infrared Light Transillumination (NILT) and optical coherence tomography, trying to employ DL algorithms for classifying carious lesions. CNNs have been employed to detect caries lesions in NILT images of extracted posterior permanent human teeth (molars and premolars), achieving an accuracy of 69%, while the sensitivity and specificity were 59% and 76%, respectively [20].

According to the literature, the most effective methods for detecting caries employing CNNs in periapical radiographs achieved diagnostic accuracy of 89% in premolars and 88% in molars [21]. Similar algorithms were used to identify cavity presence in bitewing radiography images, achieving 86.7% accuracy [22].

The integration of artificial intelligence (AI) into dental diagnostics has demonstrated significant potential in assisting clinicians with the detection and diagnosis of carious lesions from medical images. However, the clinical adoption of such technologies faces several challenges and limitations. These include variability in diagnostic performance, the invasive nature of imaging techniques that typically involve exposure to ionizing radiation, the need for extensive and high-quality datasets, substantial implementation costs, and regulatory and ethical concerns related to data privacy and algorithmic bias. The accurate, early, and precise diagnosis of dental caries is of primary importance for effective clinical management. Therefore, an ideal detection method should be non-invasive, painless, and highly reliable, with a high sensitivity rate and rapid response. Additionally, it should be applicable across all tooth surfaces and designed to preserve dental tissues and safeguard overall patient health by minimizing or avoiding exposure to ionizing radiation.

Impedance spectroscopy is a measurement technique that aligns well with the requirements for non-invasive, rapid, and sensitive detection methods, and it has already demonstrated applicability across various scientific and clinical domains. This technique, based on the measurement of electrical impedance over a range of frequencies, can be employed to measure changes in the electrical conductivity of dental tissues associated with the demineralization process. Thus, it offers a promising approach for the early diagnosis of dental caries. Demineralization leads to the breakdown of the tooth’s crystalline mineral structure, resulting in enamel that is more porous and mechanically weaker compared to healthy tissue. This degradation is primarily due to mineral loss and the consequent expansion of the inter-crystalline spaces within the enamel. Subsequently, these spaces are filled by ion-rich fluids from the oral environment, which alter the tissue’s electrical properties. Impedance spectroscopy is capable of detecting the resultant changes in conductivity, making it a valuable tool for monitoring the early stages of carious lesions [23,24].

The first studies exploring the feasibility of employing impedance spectroscopy for caries detection date back a few decades. However, the review in [25] highlighted that the diagnostic precision of various selected electrical conductance devices in comparison to standard detection methods lacks strong evidence, with only a limited number of studies focused on dental surfaces and inconsistent results that exhibit high variability. Few commercial devices are available. Among them, one of the most popular devices is CarieScan Pro™. Although the device demonstrates considerable potential, its classification accuracy remains variable, ranging from 52% to 70% [26,27], according to the age of the patient, the type of tooth, and other factors [28].

Despite the significant number of articles utilizing AI algorithms for caries detection, none to date have incorporated impedance spectroscopy measurements into their methodologies. Most of the current approaches rely on AI to classify radiographic images. However, radiographic methods inherently involve patient exposure to ionizing radiation, posing potential health risks. This paper seeks to address this gap by proposing a novel fully non-invasive diagnostic method that integrates impedance spectroscopy with a binary AI classifier to detect early-stage tooth demineralization, marking the initial phase of the carious process. A preliminary version of this work has been reported [29]. Nevertheless, in this work, two different approaches were developed, both exploiting impedance measurements, and a new device was presented and characterized. The first approach is based on a single-frequency impedance measurement carried out by an ad hoc-designed board able to perform the measurement and classification using a single-neuron binary classifier. The second approach is instead based on a complete impedance spectrum acquisition coupled to an equivalent electrical circuit fitting and a binary classification based on a neural network. The experimental validation of the two approaches was carried out on approximately one hundred impedance spectra acquired on both healthy and in vitro-demineralized teeth, obtaining very promising results. Finally, a prototype of the measurement device was presented, offering a safer and potentially more accessible alternative to traditional radiographic techniques for the detection of dental demineralization.

## 2. Sample Preparation and Data Acquisition

In order to test the proposed approaches and experimentally validate them, a set of several impedance spectra acquired on both sound and demineralized human teeth are required. The present study is based on ex vivo samples, collected at the University of Turin (see below for further details). Actually, in the last few decades, the scientific community has widely leveraged in vitro demineralization tests to investigate the fundamental processes related to mineral loss and carious progression [30].

### 2.1. In Vitro Demineralization Procedure

Demineralization can occur in any form and position on the teeth in the oral cavity. Therefore, simulating the demineralization process in a controlled environment offers a valuable approach for replicating various carious lesions, such as white spot lesions, secondary caries near restorations, caries around orthodontic brackets, and root and dentine caries. This controlled approach facilitates a faster and more consistent analysis compared to the natural progression of caries, thus enhancing experimental reproducibility and allowing for accelerated study of demineralization processes [31,32].

Although the use of human teeth is also advisable for in vitro measurements, this approach cannot account for all the environmental factors that affect tooth enamel demineralization, including tooth age, dietary habits, and pathologies, which lead to high inter- and intra-variability. With the aim to limit the measurement variability due to environmental factors, only frontal incisors extracted within one year were used for this study. In particular, fifty undamaged human front incisors were collected in the Department of Cariology and Operative Dentistry at the University of Turin in Italy, and the study protocol was approved by the University’s ethical committee (protocol DS 00071 2018). Each tooth was carefully selected by expert dentists to ensure sound samples were free from defects such as white spots, cemento-enamel defects, or dental caries that could impact the study results. Additionally, teeth had complete root formation with no dental fillings or sealants and were free from evident cracks or damage.

**Figure 1:**
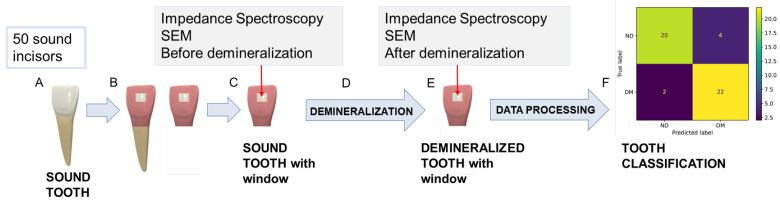
Experimental procedure carried out in this study: (**A**) tooth storage after extraction, (**B**) tooth preparation (root cut and selective window creation on the tooth surface), (**C**) first characterization of the tooth surface before demineralization, (**D**) in vitro tooth demineralization on the selected area, (**E**) second characterization after demineralization, and (**F**) data processing and tooth classification employing different classifiers.

Figure 1 shows the main steps of the experimental procedure carried out for in vitro demineralization and the subsequent data acquisition. After extraction, teeth were cleaned and stored in sealed vials with a 0.5% *w*/*v* sodium hypochlorite (NaClO) solution to prevent dehydration.

Thereafter, each tooth was sectioned and cut using a low-speed diamond saw under constant water irrigation in order to have samples of comparable size that could best adapt to the different supports used in the subsequent analyses.

In addition, to achieve a repeatable impedance spectra acquisition in the same tooth area before and after the in vitro demineralization, a selective coating of the tooth surface was carried out. Specifically, a window of 3 mm × 3 mm was isolated on the enamel surface of each tooth, while the rest of the tooth surface was covered with a protective coating, as shown in Figure 1B. The coating-free area was selected in correspondence with the central section of the third medium of the coronal vestibular face to facilitate the experimental characterizations by having a surface as flat and homogenous as possible.

A full characterization of the selected spots in terms of both surface morphology and electrical impedance was carried out in order to check if the tooth surface was sound and to acquire impedance spectra on it. Then, the exposed tooth surface was artificially demineralized using the following experimental protocol, already validated in the study [33]. Firstly, a demineralizing solution was prepared by using the following chemical components: 0.05 mol/L acetate buffer (pH 5.0), 1.28 mmol/L of Ca (CaCl_2_), and 0.74 mmol/L P (KH_2_PO_4_). Then, either sodium hydroxide (NaOH) or hydrochloric acid (HCl) was added in order to adjust pH and emulate the cariogenic condition (4.5 < pH < 5.5).

All tooth samples were then immersed in this demineralizing solution and incubated at 38 °C for 96 h without stirring. After the demineralization period, all the teeth were flushed with distilled water and stored in sealed vials containing distilled water at room temperature. Subsequently, a new full characterization of the same spots with the surfaces now demineralized was carried out to assess the demineralization state of the teeth and acquire the new impedance spectra.

In summary, the experimental procedure employed in this study involved the following steps: Fifty sound human frontal incisors were selected, sectioned, and prepared by isolating a selective window on the enamel surface for measurement. Initial surface characterization and baseline impedance spectra were recorded. The samples then underwent in vitro demineralization by immersion in a demineralizing solution at 38 °C for 96 h. Following treatment, the same enamel areas were recharacterized to evaluate the demineralization process and to acquire post-treatment impedance spectra. Finally, the collected data were analyzed using different classification algorithms. Further details on the surface characterization and impedance measurement procedures are provided in the following sections.

### 2.2. Tooth Surface Characterization

Characterization of the tooth surface was carried out in the area inside the 3 mm × 3 mm window. First, impedance measurements were performed promptly after removing the specimens from the hypochlorite solution and drying with tissue paper to ensure optimal surface conditions for measurement. Subsequently, scanning electron microscopy (SEM) images were collected in the same area with the purpose of monitoring the surface morphological changes induced by demineralization. Phenom™ XL G2 Desktop SEM (Thermo Fisher Scientific, Waltham, MA, USA) was used in this study. Since this instrument uses low-vacuum conditions, teeth were introduced into the microscope without metallization or any other surface or dehydration treatment, under controlled humidity and at room temperature.

**Figure 2:**
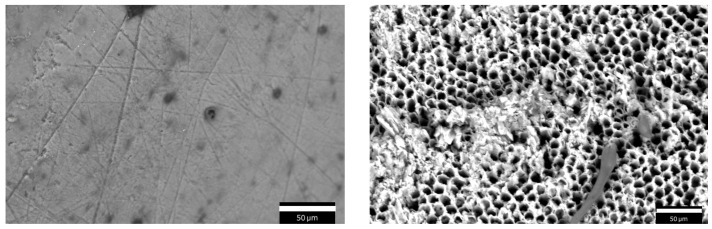
Scanning electron microscope (SEM) images of healthy enamel surface on the (**left**) and demineralized enamel on the (**right**).

SEM images were acquired using an accelerating voltage of 15 kV and a working distance of about 8.5 mm in two conditions: before the demineralization process and after the 96 h demineralization protocol. An example of the same tooth surface is shown in Figure 2 before (left) and after (right) the demineralization process. On the left, sound enamel is visible, featuring a smooth surface with some minor pits, impurities, and scratches. On the right, the enamel surface looks severely damaged due to the in vitro demineralization: enamel prism cores are dissolved and only the hexagonal structure of HA crystals is retained. As can be seen, the demineralized enamel is characterized by a porous morphology.

### 2.3. Impedance Measurements

Impedance measurements were acquired on the same spot of the tooth surface before and after the in vitro demineralization, with the goal of assessing the effects of tooth decay on the measured impedance. All measurements were carried out using the IVIUM-n-Stat potentiostat (Ivium Technologies BV, Eindhoven, The Netherlands) in a two-electrode setup.

As per common best practices in electrochemical measurements, the performance of the potentiostat is regularly checked by using the ‘dummy cell’ provided by the producer, which is composed of known electrical components.

Impedance spectra were acquired by applying a sinusoidal signal with 10mV amplitude and measuring the corresponding current in the frequency range from $10^{-1}\;\mathrm{Hz}$ to $10^{4}\;\mathrm{Hz}$, acquiring five points per frequency decade, to prevent an excessively long measurement time. Each spectrum acquisition was repeated three times to assess measurement repeatability. A 3D-printed PLA (Polylactic Acid) holder was designed to place the tooth sample inside the measurement cell and prevent any tooth damage. The sample was soaked in the electrolyte (0.9% *w*/*v* sodium chloride, NaCl, solution), leaving the upper portion of the crown exposed for measurement. For both the Working Electrode (WE) and the Counter Electrode (CE), thin platinum wires with a diameter of 0.1mm were used. The WE was placed in direct contact with the exposed enamel surface within the window, ensuring stable contact with the region of interest. The CE was immersed in the electrolyte solution.

### 2.4. Tooth Impedance Characterization and Equivalent Electrical Circuit Modeling

**Figure 3:**
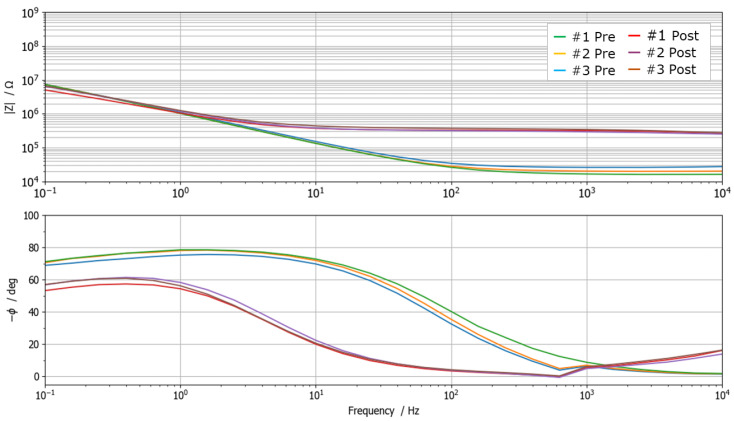
Impedance spectra collected on the same tooth sample before and after demineralization. Spectra were acquired three times to assess measurement repeatability. A distinct phase shift of approximately one frequency decade, attributable to the demineralization process, is readily observable. The negligible phase discontinuity present at 10 kHz is an instrumental artefact due to current autoranging.

Measurements highlighted a significant change in the impedance spectrum due to the demineralization process, as depicted in Figure 3. In particular, Bode diagrams show three repeated measurements carried out on the same sample before (green lines) and after (red lines) demineralization. It is possible to observe how repeated measurements are consistent and repeatable, and how the demineralization involves both a significant decrease in impedance magnitude at high frequency (increased ion concentration due to the increased porosity of the surface) and a large phase shift. All spectra exhibit resistive behavior in the high-frequency range and capacitive-like behavior at low frequencies. In particular, the high-frequency impedance response is dominated by the effect of solution resistance and, to a certain extent, by the tooth interface. Instead, the low-frequency impedance is predominantly influenced by the double-layer capacitance at the interface between the probe and the tooth surface.

**Figure 4:**
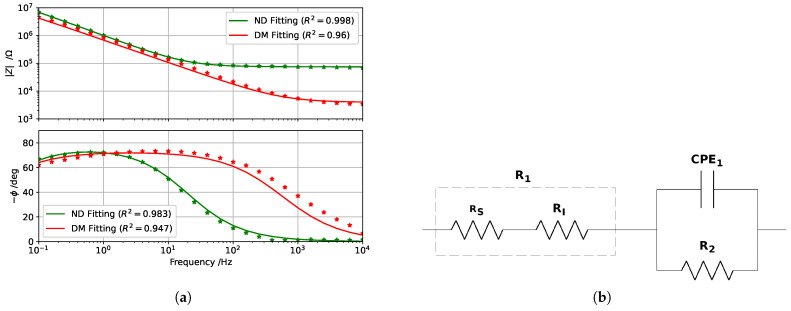
(**a**) Impedance spectra acquired before and after demineralization of the same tooth sample. Markers represent the experimental data, while the continuous line represents the equivalent electrical circuit model. (**b**) The equivalent electrical circuit used to model impedance spectra acquired on sound and demineralized teeth.

As all impedance spectra exhibited behavior that can be associated with a first-order system (i.e., one time constant), they were modeled using the equivalent electrical circuit reported in Figure 4b. The model employs three circuital elements: two resistors ($R_{1}$ and $R_{2}$) and one Constant-Phase Element (${\mathrm{CPE}}_{1}$). Such an equivalent model is extremely useful because it allows to identify how the single physical and chemical factors contribute to the impedance measurement.

The first resistor, namely $R_{1}$, takes into account contributions related to the solution resistance ($R_{S}$) and the tooth–WE interface resistance ($R_{I}$), contributions that are basically independent from the frequency. The second part of the circuit combines the double layer capacitance (${\mathrm{CPE}}_{1}$) and the charge transfer resistance ($R_{2}$). The parallel accounts for the tooth surface structure (enamel and dentin porosity and ion concentration), which is altered by demineralization [34,35]. The CPE impedance is defined as

(1) $$Z_{CPE}=\frac{1}{{\left(j\omega \right)}^{N}Q}$$

where *Q* is the CPE parameter, having the dimensions $\frac{s^{N}}{\Omega }$, *j* = $\sqrt{-1}$, and ω=2πf (*f* is the frequency), and *N* is a parameter that ranges from 0 to 1.

All acquired impedance spectra were fitted to the proposed model using the IviumSoft Software (release 4.982), which includes a complex non-linear least-square fitting algorithm. After fitting, the software provides the optimized values of the equivalent circuit elements: $R_{1}$, $R_{2}$, $Q_{1}$, and $N_{1}$.

An example of spectrum fitting is shown in Figure 4a, while the estimated element parameters, both before and after demineralization, are reported in Table 1. Even though certain variability in $R_{1}$, $R_{2}$, and $Q_{1}$ was obtained, the order of magnitude remains similar among the analyzed samples, with similar changes due to demineralization. Different behavior characterizes the $N_{1}$ parameter, whose small changes do not appear to be related to the demineralization.

## 3. Classification Algorithms

Based on the acquired data, two different approaches for the automatic classification of tooth demineralization state are proposed: a single-neuron binary classifier and an MLP neural network. Despite the relatively limited size of the dataset, it is important to note that obtaining large datasets when using human tissues is inherently challenging. In this specific case, the requirement for the collected teeth to be sound and undamaged further complicates the acquisition of a large sample size. Nevertheless, the authors are confident that the data quantity is sufficient to substantiate the conclusions drawn in this study. A review of the relevant scientific literature reveals that the number of samples used is comparable to similar studies [36,37,38], which validates the adequacy of the dataset size for this research.

### 3.1. Single-Neuron Classifier

The first investigated classification approach takes advantage of the large magnitude and phase shifts detected in the impedance spectra acquired before and after the in vitro demineralization process (Figure 3). Such large changes in the spectrum can be exploited in order to discriminate between sound and demineralized teeth. In particular, the algorithm employs a single-phase measurement of tooth impedance at the frequency of 15Hz as input parameter for classification. In fact, preliminary investigations [29,39] highlighted how phase measurements are more reliable than magnitude ones due to an intrinsic lower sensitivity to tooth shape and size, electrode contact, and external noise interference. Moreover, the same study showed that, on average regarding the investigated dataset, the highest phase shift occurs at a frequency of 15Hz.

**Figure 5:**
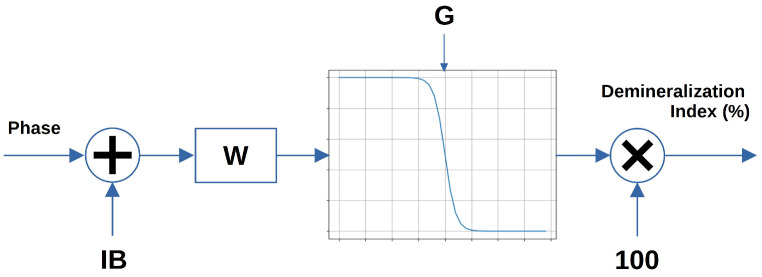
Block diagram of the single-neuron classifier.

The proposed classifier is based on a single perceptron that employs an inverted sigmoid as decision function. It takes the impedance phase at 15Hz as input and returns the tooth demineralization index ranging from 0% for sound teeth to 100% for totally demineralized teeth. To discriminate between these two scenarios, a threshold of 50% was employed. Figure 5 shows the block diagram of the classifier.

The classification parameters that can be tuned are Input Bias (IB), Weighting Factor (W), and sigmoid gain (G). These parameters were configured to minimize the classifier’s error rate over the training dataset with the aim of relating them to their statistical meaning. Firstly, IB was set to 65, derived from the average phase value of demineralized teeth and adding 1.5 times the phase standard deviation. G determines the smoothness of the decision function, with higher G values involving a steeper function. The lowest error rate is achieved with a gain value of 2. Finally, W was set to 1 in order to avoid alterations to the input phase.

**Figure 6:**
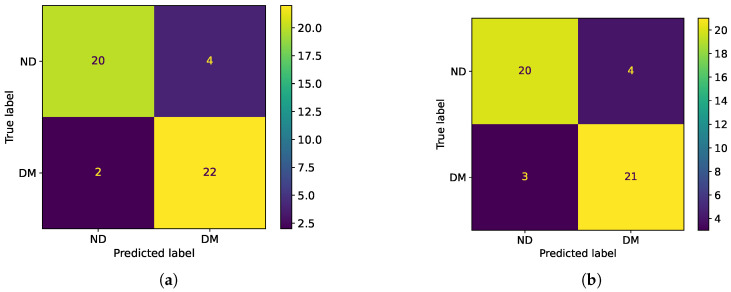
(**a**) Classification results of the single-neuron binary classifier over a balanced dataset of 24 teeth, shown as confusion matrix. (**b**) Classification results of the MLP classifier using two hidden layers, respectively, of three and two neurons, shown as a confusion matrix. In both plots, labels ND and DM stand for “Non-Demineralized” and “Demineralized” teeth, respectively.

A different set of impedance spectra were used to validate the performance of the proposed classifier. Figure 6a shows the confusion matrix obtained by the balanced test dataset composed of 24 sound teeth that were artificially demineralized according to the described procedure. Table 2 reports the principal classification scores achieved by the single-neuron classifier.

### 3.2. MLP Classifier

A quite different approach is instead employed in the second classifier. After fitting the equivalent circuital model, as described in Section 2.4, a preliminary analysis of the electrical parameters highlighted a change in their values that could be related to the demineralization state of the samples. Thus, to assess if such variation can be exploited for tooth state classification, an MLP classifier was designed by using the Python module scikit-learn (version 1.2.2) [40].

In order to perform a comparison between the two proposed approaches, training, validation, and testing of the neural network were carried out by using the same dataset, randomly splitting data in separate groups: 70% for training and 30% for testing. Several tests and training–validation cycles were carried out employing 5-fold cross-validation and trying to obtain the best-performing network topology while also optimizing the training parameters. Tests with different combinations of input parameters ($R_{1}$, $R_{2}$, $Q_{1}$, and $N_{1}$) showed that the performance of the classifier degraded when including the parameter $N_{1}$. For this reason, it was not included in the subsequent computations.

The best performance was achieved with a neural network employing two hidden layers, respectively composed of three and two neurons, with all neurons using hyperbolic tangent as activation function. Parameters $R_{1}$, $R_{2}$, and $Q_{1}$ were used as input for each tooth sample. Due to the high difference in magnitude of these parameters, a proper normalization was carried out before feeding the network. Among the different normalization schemes, the best performance was achieved with a standard “min–max” normalization. The algorithm “L-BFGS” (Limited-Memory Broyden–Fletcher–Goldfarb–Shanno algorithm), with an adaptive learning rate (α=0.0001), was employed as a solver for neuron weight optimization because it appeared to perform better on the small dataset available. Classification performance during the 5-fold cross-validation in the final network configuration is reported in Table 3, which highlights a quite uniform performance distribution across the different splits.

Finally, the network was tested using the test dataset. The results achieved are comparable with the single-neuron classifier, as evident from the confusion matrix shown in Figure 6b and the scores reported in Table 2.

**Figure 7:**
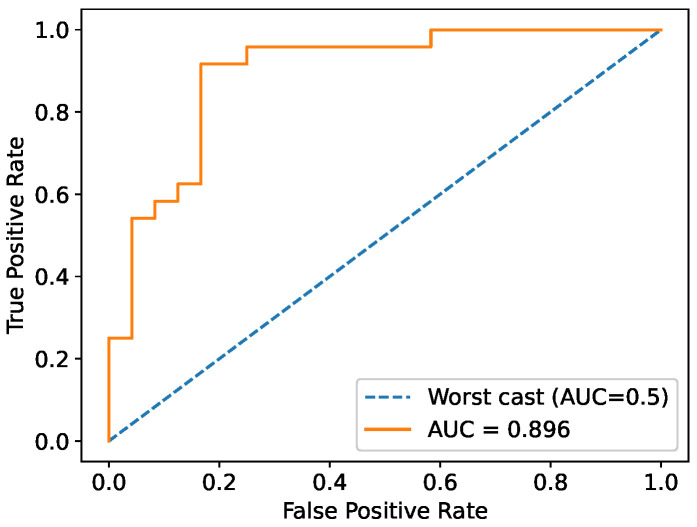
ROC–AUC curve for the MLP classifier on the test dataset, showing very good capability of the classifier to discriminate between demineralized and non-demineralized teeth. The dashed line represents the worst case corresponding to random behavior of the classifier.

Additionally, the ROC (receiver operating characteristic) curve was computed and the AUC was estimated to be 0.894, as reported in Figure 7. In particular, the AUC values are very close to 1, indicating an excellent capability of the classifier to discriminate between the two tooth classes (demineralized and non-demineralized).

Once the MLP classifier was trained, the processing time was estimated, resulting in an average of 200 ms on a middle-range laptop (Processor: AMD^®^ Ryzen 5 4500U, RAM: 8 GB, OS: Ubuntu 22.04 LTS, Python 3.10.12).

### 3.3. Classification Results

The two proposed classifiers perform in a similar way on the tested dataset, with results that are promising, as demonstrated in the two confusion matrices (Figure 6a,b) and by the classification scores reported in Table 2. In particular, the accuracy achieved by the two classifiers is quite high, reaching 0.88 and 0.85, respectively. Due to the use of a balanced dataset, accuracy and F1-score are very close to each other. Moreover, the classifiers perform well both on demineralized and non-demineralized teeth, as is possible to note from the symmetry of the confusion matrices and the similarity of the recall and specificity scores. Even though both classifiers are based on tooth surface impedance measurements, they use different input data. Indeed, the MLP algorithm needs three parameters, namely $R_{1}$, $R_{2}$, and $Q_{1}$, which are obtained by the equivalent circuit fitting, and thus a complete impedance spectrum has to be acquired to perform the classification. So, this represents a significant limitation for the implementation of a diagnostic tool for two main reasons. First, it increases the measurement time and system complexity (if compared to a single-phase measurement); then, after impedance spectrum acquisition, a fitting algorithm is required to obtain the equivalent circuit parameters. For these reasons, the approach based on single neurons demonstrates to be the most appropriate one to implement a non-invasive portable device for tooth demineralization assessment.

## 4. Prototype of the Measurement Device

Due to the considerations regarding the two classifiers, the authors decided to employ the single-neuron classifier for the development of an automatic diagnostic measurement system able to assess the demineralization state of a tooth.

The development was carried out taking into consideration the following requirements:

- the device should be portable and characterized by a small dimension so that it can be easily handled and is not an obstacle for either the dentist or the patient;
- battery powered, featuring, therefore, low power consumption;
- easy to be employed and able to provide an easy-to-understand result;
- safe for the patient, non-invasive, and able to perform the measurement in a few seconds.

### 4.1. Prototype Implementation

**Figure 8:**
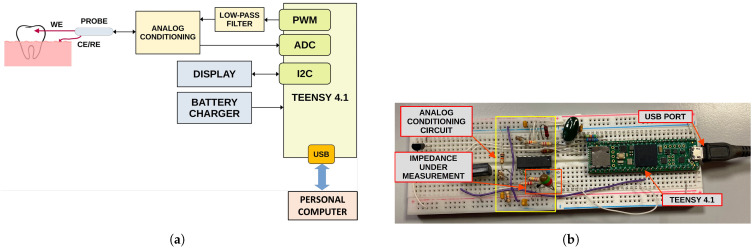
(**a**) Block diagram of the developed prototype for the automatic assessment of tooth demineralization. Blocks in gray color are not implemented yet. (**b**) Photo of the developed prototype based on a Teensy 4.1 and few external components. The prototype is able to measure the impedance phase at a fixed frequency and classifies the tooth state by using the single-neuron classifier.

The prototype is arranged around the Teensy 4.1 (PJRC, Sherwood, Portland, OR, USA), a small commercial development board that embeds a powerful 600MHz 32-bit ARM Cortex-M7 microcontroller, 8MB of RAM, and several other peripherals. A 16-bit unipolar Analog-To-Digital Converter (ADC) is available as well for directly acquiring analog signals. The block diagram of the system is shown in Figure 8a. The system implements both the impedance measurement and tooth classification, aiming to employ the lowest number of external components as possible in order to reduce the cost and the size of the device.

Impedance measurement is carried out at a single frequency, which can be selected between 1Hz and about 100Hz by generating a sinusoidal voltage of a few tens of millivolts (stimulus) and acquiring the corresponding current. Unfortunately, Teensy 4.1 does not have a Digital-To-Analog Converter (DAC); therefore, the stimulus signal is generated by filtering a proper PWM signal with a first-order low-pass filter. Alternatively, a Teensy 3.6, which embeds a DAC, can be employed.

A dedicated analog conditioning circuit based on a quadruple operational amplifier (TL084, Texas Instruments, Dallas, TX, USA) is employed to shift and buffer the generated stimulus as well as to acquire and amplify the current signal using a trans-impedance amplifier.

After raw data acquisition is performed, a non-linear fitting of the sinusoidal current is performed with the aim of estimating the frequency, amplitude, and phase of the acquired current with respect to the generated voltage. Such a fitting approach improves the accuracy of the phase measurement and removes any residual high-frequency harmonic due to the PWM stimulus. Finally, the magnitude and phase of the impedance are calculated. In order to further reduce the effects of noise, 10 phase measurements are carried out in sequence, and the average value is then employed for classification.

Impedance phase value is thereafter fed to the single-neuron classifier, implemented in the microcontroller firmware directly in C Language. The classification result, provided as percentage of demineralization, together with the raw data, are finally sent over the USB port to the host PC for analysis and system validation. A photo of the developed prototype is shown in Figure 8b. A display for the direct visualization of the classification result will be added in the future, together with a Li–Po battery charger, aiming to realize a fully portable device. At the moment, no experimental test for the battery operative life has been carried out. However, a first rough estimation can be easily performed. Powering the device from a single-cell lithium-ion rechargeable battery with a capacity of 400 mAh allows a compact implementation of the system and provides sufficient operative life. The power consumption of the Teensy 4.1 board is in the order of 200 mA at a medium computing load and includes a low-power analog front-end and a 1″ OLED display. Therefore, considering that each measurement requires about 2 s to complete and about 28 s for device management and result visualization, it is possible to estimate the operative life in about 240 measurements with a single battery charge. However, battery self-discharge and device usage can reduce operative life. Nevertheless, the possibility of recharging the device battery from a common USB interface makes it suitable for portable applications.

The firmware was written in C (Arduino IDE, version 2.3). Raw data are directly acquired using the integrated ADC, while the stimulus sine signal is generated using an LUT (Look-Up Table) and the Timer PWM (Pulse Width Modulation) implemented in the firmware. The algorithm is extremely efficient and does not require high computing resources. Data acquisition and sine non-linear fitting require about 2 s, while the classification with the single neuron takes less than 20 ms, which is negligible with respect to the acquisition time.

### 4.2. Preliminary System Validation

A preliminary validation of the developed prototype was carried out in order to assess if the proposed system is able to measure impedance (magnitude and phase) at a suitable level of accuracy for the subsequent classification algorithm.

**Figure 9:**
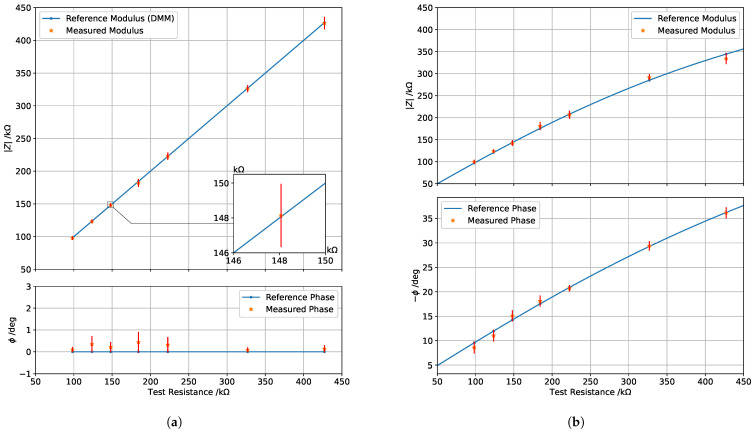
Preliminary characterizations of the prototype in terms of measurement accuracy of impedance modulus and phase: (**a**) for pure resistors in the range from 100 kΩ to 500 kΩ; (**b**) for an R–C parallel circuit obtained with a fixed capacitor of 18.2 nF while changing the resistor value between 100 kΩ and 500 kΩ.

Impedance spectra acquired on the samples highlighted that the expected impedance of a tooth at 15 Hz is in the range from 100 kΩ to 500 kΩ both for demineralized and non-demineralized teeth. Therefore, preliminary system validation was carried out in this range. First, known resistors, with values taken in the above range, were measured with the prototype and a reference bench multimeter (HP, model 34401A). The results, shown in Figure 9a, confirm the good performance of the device, which achieves a maximum impedance modulus uncertainty of about 9 kΩ and a maximum impedance phase uncertainty of about 0.6°.

Subsequently, an additional test was carried out to measure the impedance of an R–C parallel circuit, where a capacitor of about 18.2 nF was kept fixed while changing the resistor value between 100 kΩ and 500 kΩ. The results achieved are reported in Figure 9b. In particular, the blue line represents the theoretical expected impedance (modulus and phase) estimated by the measured values of R and C, while the single samples are the impedance values measured by the prototype. Values of phase are considered always positive for simplicity, this prototype being intended to measure only impedance on teeth, which always exhibit capacitive behavior. It is possible to see how the results are very promising with prototype measurements very close to the expected ones both for impedance modulus and phase, even though uncertainty slightly increased with respect to pure resistors due to the effect of capacitance on the analog circuit. In particular, the maximum uncertainty on the impedance modulus increased to about 15 kΩ, while the maximum uncertainty on phase reached about 2°. Nevertheless, the metrological performance is more than suitable for tooth classification, with the achieved uncertainty being lower than the intrinsic natural variability of tooth impedance, as highlighted by the spectra acquired on the samples.

## 5. Conclusions

This study demonstrates the feasibility of using impedance spectroscopy to distinguish between healthy and demineralized teeth, enabling a non-invasive, radiation-free methodology for carious lesion detection. A dataset of over 100 samples was collected, acquiring impedance spectra before and after an in vitro demineralization process. By analyzing the impedance spectra, significant variations in modulus and phase were identified and correlated with the degree of surface demineralization. A first-order equivalent circuit model showed how circuit element values depend on tooth condition.

Two binary classifiers were developed to automatically assess tooth demineralization. A single-neuron classifier, operating on a single impedance phase measurement at 15 Hz, achieved 88% accuracy and offers a lightweight, low-power solution that is suitable for microcontroller implementation. This approach simplifies the electronics and avoids the need to acquire a full impedance spectrum, thus reducing measurement time. A second classifier, based on an MLP neural network, utilized equivalent circuit element values and achieved a similar accuracy of 86%. While both performed well, the single-neuron classifier is more practical for clinical use due to its simplicity and efficiency.

A prototype system was developed based on the single-neuron approach using a Teensy 4.1 microcontroller to measure the impedance phase and classify tooth states. The preliminary validation indicated a maximum impedance phase uncertainty below 2°, supporting the reliability of the classification algorithm.

The performance of the presented classifiers meets or exceeds that of the existing methodologies employing machine learning and deep learning algorithms for dental caries detection and diagnosis. The proposed impedance-based method offers several potential clinical advantages over the traditional techniques, i.e., visual–tactile tools and radiography, which are invasive, operator-dependent, and entail patient exposure to ionizing radiation. In contrast, impedance-based detection is inherently safe, rapid, repeatable, and painless, rendering it particularly advantageous for vulnerable patient populations. The integration of AI with tooth impedance spectra represents a novel approach in dental diagnostics as the majority of the current AI-driven methods focus on the classification of radiographic images. This study addresses this research gap by introducing a fully non-invasive diagnostic modality for an early, rapid, painless, and reliable alternative with high sensitivity for early caries detection.

Despite the promising results, this in vitro study presents inherent limitations. Firstly, all the data were collected in vitro on the same kind of teeth (frontal incisors) that were sound and undamaged prior to the induced demineralization process. This homogeneity in the sample population restricts the generalizability of our findings as it does not account for the biological variability observed in vivo, including factors such as patient age, dietary habits, the complex oral environment, and pathology-related variations. Secondly, the electrochemical measurements were conducted in a controlled environment under repeatable conditions (i.e., temperature and humidity in the laboratory). This controlled environment, while ensuring repeatability, does not fully replicate the fluctuating conditions encountered in the oral cavity. Future research should aim to validate these findings using a more diverse sample population and under conditions that more closely mimic the dynamic in vivo environment.

To address the identified limitations and further validate the proposed methodology, future research will focus on (1) expanding the dataset to include diverse tooth types with varying conditions and pathologies; (2) developing a biocompatible and sterilizable in vivo probe for direct intraoral measurements; and (3) conducting clinical trials to evaluate the system’s performance under real-world conditions. Efforts will also target the design of an intuitive user interface and cost-effective system integration for practical clinical adoption. The combination of impedance spectroscopy and AI-based classification offers a novel contribution to the field of dental diagnostics, providing a compelling non-invasive alternative to prevalent radiographic AI applications and addressing a critical research gap. The developed prototype system holds the potential to improve early caries management through objective readily interpretable diagnostic information, potentially reducing reliance on operator expertise and the need for invasive or costly treatments. This research lays a foundation for the development of practical tools for early, accessible, and accurate dental diagnostics.

## Figures and Tables

**Table 1:** Values of the equivalent circuit elements estimated by fitting the impedance spectra before and after demineralization.

	Demineralized Samples	Non-Demineralized Samples
$R_{1}$ (Ω)	$7.5\times 10^{4}$	$3.9\times 10^{3}$
$R_{2}$(Ω)	$3.4\times 10^{7}$	$2.6\times 10^{7}$
$N_{1}$	0.86	0.81
$Q_{1}$ ($s^{N_{1}}/\Omega$)	$2.1\times 10^{-7}$	$3.3\times 10^{-7}$

**Table 2:** Comparison between the classification scores achieved by the single-neuron classifier and MLP classifier over the same balanced test dataset of 24 teeth.

Score	Single-Neuron Classifier	MLP Classifier
Accuracy	0.88	0.85
F1-Score	0.88	0.86
Recall	0.92	0.88
Specificity	0.83	0.83
Precision	0.85	0.84
Negative Predictive Value	0.91	0.87

**Table 3:** Five-fold cross-validation performance of the MLP classifier over the 5 splits and the average performance.

Cross-Validation	Accuracy	Recall	AUC
Split #1	0.83	0.8	0.83
Split #2	0.91	1	0.87
Split #3	0.73	0.8	0.77
Split #4	0.82	0.7	0.93
Split #5	0.91	1	0.91
Average	0.84	0.86	0.86
Standard Deviation	0.07	0.12	0.06

## Data Availability

Data will be made available upon request.
